# Evidence for a Lean Mass Hyperresponder Phenotype Is Lacking with Increases in LDL Cholesterol of Clinical Significance in All Categories of Response to a Carbohydrate-Restricted Diet

**DOI:** 10.1093/cdn/nzac043

**Published:** 2022-05-27

**Authors:** Jeff M Moore, Dominik Diefenbach, Makarand Nadendla, Nicholas Hiebert

**Affiliations:** School of Exercise and Nutritional Sciences, San Diego State University, San Diego, CA, USA; Witten/Herdecke University, Germany; Independent Researchers; Independent Researchers

Dear Editor:

In “Elevated LDL cholesterol with a carbohydrate-restricted diet: evidence for a ‘lean mass hyper-responder’ phenotype” (1) the authors aim to explain sources of heterogeneity in LDL cholesterol changes resulting from a carbohydrate-restricted diet (CRD) to identify individuals at risk of such changes. Several issues within this publication are apparent including those related to methodology and interpretation. These issues lead to erroneous and potentially harmful conclusions.

## Methods

**FIGURE 1:**
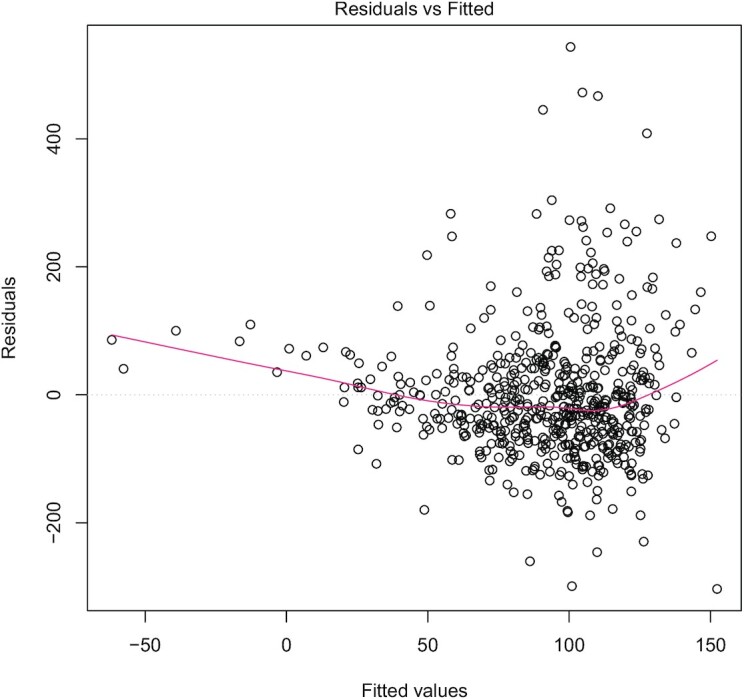
Residual plot of BMI and prior TG:HDL cholesterol ratio predicting change in LDL cholesterol. The typical cone shape of the points, curved regression of residuals on fitted values (red line), and skewed distribution of the residuals show that without further transformation of the data linear regression via ordinary least squares is likely inappropriate. TG, triglyceride.

**FIGURE 2:**
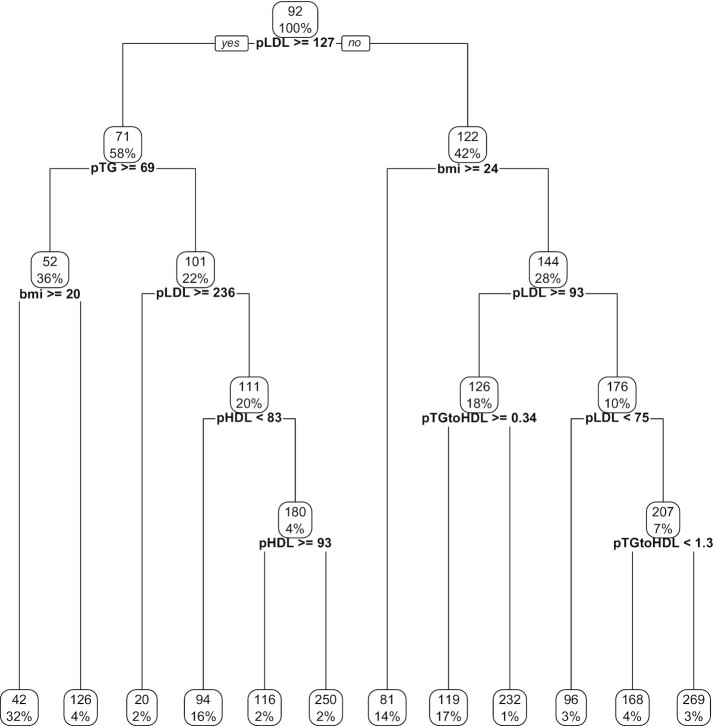
Decision tree of LDL cholesterol change limited to variables used by Norwitz et al. (1), including prior LDL cholesterol. Percentages represent the proportion of participants discriminated at each node. BMI, body mass index; pLDL, prior LDL cholesterol; pTG, prior triglycerides; pHDL, prior HDL; pTGtoHDL, ratio of prior triglycerides to prior HDL.

1. The methodology is insufficient to address the purpose of the study. Whereas BMI, sex, age, and all prior lipid markers were included, larger sources of known heterogeneity, including dietary components not ascertained in the original survey and genetics, are not. Exclusion criteria likely contributed to selection bias, and the platform of distribution to participation bias. Those with higher BMI and/or TG:HDL cholesterol ratio would be more likely to visit their physician and/or be recommended lipid-lowering medication. Although this limitation is addressed, the purpose is better described as examining the effect of the limited number of variables on LDL cholesterol changes.
2. Use of TG:HDL cholesterol ratio to suggest cardiometabolic health requires justification, particularly in the context of a CRD for which associations between TG:HDL cholesterol and hard endpoints are lacking. Unlike apoB, TG:HDL cholesterol is not an established independent causal factor for cardiometabolic risk (2).
3. BMI is used to measure leanness. BMI measures the risk of obesity, not body fat. “Normal BMI hyperresponder” (NBHR) is a more accurate term for the phenotype identified. Furthermore, leanness is not defined. Case #2 had a body fat percentage of 22.5%, which is arguably not lean for a female but within normal range. If terminology is corrected to the more accurate “NBHR,” BMI could be used to develop categories for the supposed phenotype.
4. Justification for excluding prior LDL cholesterol in evaluating the source of heterogeneity is not given, yet the authors:
  - Did not evaluate how prior LDL cholesterol associated with LDL cholesterol change in their linear regression models (Table 2 in Norwitz et al.) despite performing linear regressions for all lipid and anthropometric factors (Supplemental Table 1 in Norwitz et al.) (1). When prior LDL cholesterol was included in our reanalysis of their data, constrained to the survey variables the authors included in their paper, 4 models outperforming BMI and TG:HDL cholesterol were produced (**Table 1**). Including other survey variables that influence LDL cholesterol (fat and net carbohydrate intake) (3, 4), we found 5 more models outperforming BMI and prior TG:HDL cholesterol (**Table 2**). Whether a linear regression is appropriate in the first place is in question (**Figure 1**).
  - Did not include prior LDL cholesterol as an input variable for the algorithm producing the hypothesis-naïve decision tree of LDL cholesterol change (Supplemental Figure 3 in Norwitz et al.). (1) When included in our reanalysis, the algorithm frequently selected prior LDL cholesterol at different cutoffs (**Figure 2**), preferential to any other variable.

**TABLE 1:** Predictors of changes in LDL cholesterol following a CRD limited to variables used by Norwitz et al. (1)^1^

Model		*R* ^2^	Adjusted *R*^2^	β	SE	95% CI lower bound	95% CI upper bound	*P*
Model 1	Intercept	0.131	0.128	331.86	27.01	278.93	384.79	2.00E-16
	Prior LDL cholesterol			−7.28	1.01	−9.26	−5.30	1.77E-12
	BMI			−0.44	0.07	−0.58	−0.31	1.13E-10
Model 2	Intercept	0.091	0.088	99.50	15.50	69.11	129.88	2.81E-10
	Prior LDL cholesterol			−0.47	0.07	−0.60	−0.33	5.14E-11
	Prior HDL cholesterol			0.96	0.20	0.57	1.35	1.69E-06
Model 3	Intercept	0.088	0.0854	176.61	12.02	153.05	200.16	2.00E-16
	Prior LDL cholesterol			−11.41	2.46	−16.24	−6.59	4.41E-06
	Prior TG:HDL cholesterol			−0.44	0.07	−0.57	−0.30	6.68E-10
Model 4	Intercept	0.082	0.079	178.24	12.52	153.70	202.77	2.00E-16
	Prior LDL cholesterol			−0.42	0.07	−0.55	−0.28	3.06E-09
	Prior TG			−0.26	0.06	−0.38	−0.14	4.14E-05
Model 5	Intercept	0.073	0.07	242.50	26.40	190.00	294.00	2.00E-16
	Prior TG:HDL cholesterol			−4.50	2.70	−9.81	0.70	0.09
	BMI			−5.90	1.10	−8.20	−3.70	2.70E-07
Model 6	Intercept	0.037	0.033	68.50	18.10	33.00	104.00	1.70E-04
	Prior HDL cholesterol			0.60	0.22	0.17	1.04	0.007
	Prior TG			−0.17	0.07	−0.30	−0.03	0.015

1 CRD, carbohydrate-restricted diet; TG, triglyceride; E, x 10^x^e.g. 1.70E-04 = .00017

**TABLE 2:** Predictors of changes in LDL cholesterol following a CRD not limited to variables used by Norwitz et al. (1)^1^

Model		*R* ^2^	Adjusted *R*^2^	β	SE	95% CI lower bound	95% CI upper bound	*P*
Model 7	Intercept	0.117	0.114	285.03	25.19	235.66	334.40	2.00E-16
	BMI			−6.9	1.02	−8.90	−4.90	2.76E-11
	Net carbs			−0.98	0.17	−1.31	−0.65	1.49E-08
Model 8	Intercept	0.113	0.11	187.29	12.06	163.65	210.93	2.00E-16
	Prior LDL cholesterol			−0.45	0.07	−0.59	−0.31	1.07E-10
	Net carbs			−1.07	0.17	−1.40	−0.74	8.94E-10
Model 9	Intercept	0.078	0.075	231.59	27.41	177.87	285.31	2.29E-16
	BMI			−6.79	1.04	−8.83	−4.75	1.38E-10
	Fat			0.18	0.07	0.04	0.32	0.013
Model 10	Intercept	0.074	0.071	136.82	7.69	121.75	151.89	2.00E-16
	Prior TG:HDL cholesterol			−9.99	2.48	−14.85	−5.13	6.30E-05
	Net carbs			−0.95	0.18	−1.30	−0.60	7.61E-08
Model 11	Intercept	0.073	0.07	142.94	8.72	125.85	160.03	2.00E-16
	Prior TG			−0.25	0.06	−0.37	−0.13	8.50E-05
	Net carbs			−0.97	0.18	−1.32	−0.62	5.19E-08

1 CRD, carbohydrate-restricted diet; TG, triglyceride; E, x 10^x^e.g. 1.70E-04 = .00017

### Interpretations

1. Low prior TG:HDL cholesterol and low BMI are stated to have a strong association with LDL cholesterol changes but have an adjusted *R*^2^of 0.07, which is more appropriately described as very small (5), small (6), or inadequate (7). Meanwhile, in our reanalysis, an adjusted *R*^2^ of 0.13 for prior LDL cholesterol and BMI could be interpreted as small, medium, or adequate.
2. It is concluded that individuals with obesity “may be at low risk of experiencing a clinically significant increase in LDL cholesterol with this dietary intervention.” In the highest BMI quartile, an LDL cholesterol increase of 35 to 44 mg/dL was observed. This magnitude is associated with a 20% increased risk of coronary artery disease (CAD) over 5 y (8). Suggesting that LDL cholesterol increases associated with a 20% increased risk of CAD are not of clinical significance is irresponsible and almost certain to cause harm if heeded. According to the 2018 American Heart Association Guideline on the Management of Blood Cholesterol, for 87 (87%) and 273 (61%) of LMHRs and non-LMHRs from the survey “maximally tolerated statin therapy is recommended” for the purpose of primary atherosclerotic cardiovascular disease (ASCVD) prevention (9).
3. The authors continue by referencing a study wherein the induction of a CRD was commensurate with an improvement in many ASCVD risk markers (10). It is unclear if this study is consistent with the wider literature on CRDs and LDL cholesterol, because the CRD was particularly high in cheese, a high-SFA food known for not significantly increasing LDL cholesterol. Whether there is a clinical benefit to improving other ASCVD risk markers at the expense of clinically significant increases of LDL cholesterol in response to a CRD, is unclear and requires supporting evidence.
4. In Supplemental Figure 5 in Norwitz et al. (1) directed acyclic graphs (DAGs) are proposed to exclude heterogeneity in LDL cholesterol change being explained by differential SFA intake by LMHRs and non-LMHRs. Although limitations inherent to the study like participation bias, selection bias, or chance could contribute to different SFA intakes, the DAGs have multiple issues:
  - These DAGs are 2 of many possible DAGs one could formulate in this context, many of which would include potentially causal variables not assessed in the survey.
  - In DAGs, arrows indicate causality, which implies temporality. The arrow in panel A from “↓carbohydrate intake” to “↑baseline BMI” is interpreted as “lowering carbohydrate intake causes a previously higher BMI.”

An alternative conclusion is as follows. The effect size of the correlation between BMI and TG:HDL cholesterol and changes in LDL cholesterol on a CRD is very small, small, or inadequate. Changes in LDL cholesterol following a CRD would be associated with an increased risk of CAD of ∼20% over 5 y in the highest BMI and highest TG:HDL cholesterol quartiles, and an increased risk of CAD of >40% over 5 y in the lowest BMI and lowest TG:HDL cholesterol quartiles, suggesting clinically significant increased risk regardless of BMI and TG:HDL cholesterol. Evidence supporting an LMHR phenotype is weak but might suggest that those with lower BMI and lower TG:HDL cholesterol are at even greater risk of clinically significant changes in LDL cholesterol. Alternatively, with 93% of the variance in LDL cholesterol changes on a CRD unexplained by BMI and prior TG:HDL cholesterol, other variables including diet, genetics, and behavior are necessary to elucidate heterogeneous LDL cholesterol responses to a CRD. This elucidation is greatly needed because the clinical risk is apparent.

## Notes

Author disclosures: NH is the founder of the Nutri-Dex and Nutrivore podcast for which he receives financial donations (e.g., through Patreon). All other authors report no conflicts of interest.

The authors reported no funding received for this study.

Author contributions—JM, DD, MN, and NH: designed research (project conception, development of overall research plan, and study oversight). MN: analyzed data or performed statistical analysis. JM, DD, MN, and NH: wrote paper (only authors who made a major contribution). JM, DD, MN, and NH: had primary responsibility for final content. All authors have read and approved the final manuscript.
